# Peripheral blood mitochondrial DNA copy number as a predictor of steatotic liver disease development: insights from epidemiological and experimental studies

**DOI:** 10.1265/ehpm.25-00025

**Published:** 2025-05-28

**Authors:** Genki Mizuno, Atsushi Teshigawara, Hiroya Yamada, Eiji Munetsuna, Yoshiki Tsuboi, Yuji Hattori, Mirai Yamazaki, Yoshitaka Ando, Itsuki Kageyama, Takuya Wakasugi, Naohiro Ichino, Keisuke Osakabe, Keiko Sugimoto, Ryosuke Fujii, Hiroaki Ishikawa, Nobutaka Ohgami, Koji Ohashi, Koji Suzuki

**Affiliations:** 1 Department of Medical Technology, Tokyo University of Technology School of Health Sciences, 5-23-22 Nishi-Kamata, Ota, Tokyo 144-8535, Japan; 2 Department of Preventive Medical Sciences, Fujita Health University School of Medical Sciences, 1-98 Dengakugakubo, Kutsukake-cho, Toyoake, Aichi 470-1192, Japan; 3 Department of Joint Research Laboratory of Clinical Medicine, Fujita Health University Hospital, 1-98 Dengakugakubo, Kutsukake-cho, Toyoake, Aichi 470-1192, Japan; 4 Department of Hygiene, Fujita Health University School of Medicine, 1-98 Dengakugakubo, Kutsukake-cho, Toyoake, Aichi 470-1192, Japan; 5 Department of Animal Science and Biotechnology, Azabu University School of Veterinary Medicine, 1-17-71 Fuchinobe, Chuo-ku, Sagamihara, Kanagawa 252-5201, Japan; 6 Department of Informative Clinical Medicine, Fujita Health University School of Medical Sciences, 1-98 Dengakugakubo, Kutsukake-cho, Toyoake, Aichi 470-1192, Japan; 7 Department of Clinical Physiology, Fujita Health University School of Medical Sciences, 1-98 Dengakugakubo, Kutsukake-cho, Toyoake, Aichi 470-1192, Japan

**Keywords:** Mitochondria, Mitochondrial DNA-copy number, Steatotic liver disease, Peripheral blood, Predictive marker

## Abstract

**Background:**

Mitochondria, which harbor their own genome (mtDNA), have attracted attention due to the potential of mtDNA copy number (mtDNA-CN) as an indicator of mitochondrial dysfunction. Although mtDNA-CN has been proposed as a simple and accessible biomarker for metabolic disorders such as metabolic dysfunction-associated steatotic liver disease, the underlying mechanisms and the causal relationship remain insufficiently elucidated. In this investigation, we combined longitudinal epidemiological data, animal studies, and in vitro assays to elucidate the potential causal relationship between reduced mtDNA-CN and the development of steatotic liver disease (SLD).

**Methods:**

We conducted a longitudinal study using data from a health examination cohort initiated in 1981 in Yakumo, Hokkaido, Japan. Data from examinations performed in 2015 and 2022 were analyzed, focusing on 76 subjects without SLD at baseline (2015) to assess the association between baseline mtDNA-CN and subsequent risk of SLD development. In addition, 28-day-old SD rats were fed ad libitum on a 45% high-fat diet and dissected at 2 and 8 weeks of age. Blood and liver mtDNA-CN were measured and compared at each feeding period. Additionally, in vitro experiments were performed using HepG2 cells treated with mitochondrial function inhibitors to induce mtDNA-CN depletion and to examine its impact on intracellular lipid accumulation.

**Results:**

Epidemiological analysis showed that the subjects with low mtDNA-CN had a significantly higher odds ratio for developing SLD compared to high (odds ratio [95% confidence interval]: 4.93 [1.08–22.50]). Analysis of the animal model showed that 8 weeks of high-fat diet led to the development of fatty liver and a significant decrease in mtDNA-CN. A further 2 weeks of high-fat diet consumption resulted in a significant decrease in hepatic mtDNA-CN, despite the absence of fatty liver development, and a similar trend was observed for blood. Complementary in vitro experiments revealed that pharmacologically induced mitochondrial dysfunction led to a significant reduction in mtDNA-CN and was associated with increases in intracellular lipid accumulation in HepG2 cells.

**Conclusions:**

Our findings suggest that reduced mtDNA-CN may contribute causally to SLD development and could serve as a convenient, noninvasive biomarker for early detection and risk assessment.

**Supplementary information:**

The online version contains supplementary material available at https://doi.org/10.1265/ehpm.25-00025.

## Graphical Abstract

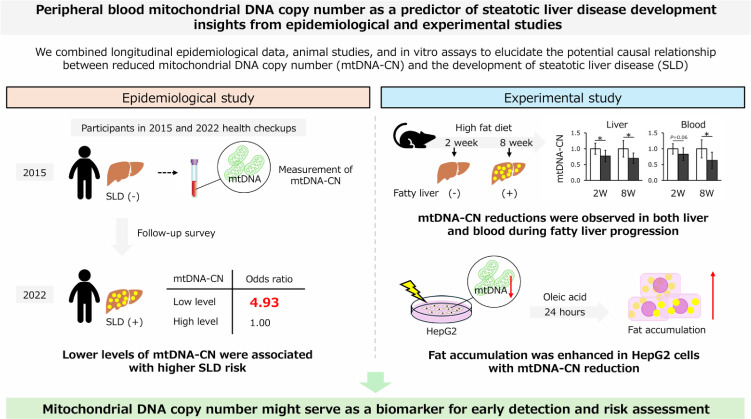

## Introduction

Steatotic liver disease (SLD) is a condition characterized by the accumulation of fat in the liver, primarily due to sedentary lifestyles and unhealthy diets. Affecting approximately a quarter of the global adult population, SLD pose a significant health and economic burden worldwide [1]. While most cases are asymptomatic, untreated SLD can progress to chronic liver diseases or increase the risk of other lifestyle-related conditions [2, 3]. In Japan, over 20 million individuals are affected by metabolic dysfunction-associated steatotic liver disease (MASLD), and this number is expected to rise further [4, 5]. Thus, the prevention and early detection of fatty liver represents a critical global health challenge.

Mitochondria possess their own genome, known as mitochondrial DNA (mtDNA). The mitochondrial DNA-copy number (mtDNA-CN), which measures the number of mitochondrial genomes per cell, is recognized as a biomarker of mitochondrial dysfunction and is strongly correlated with energy metabolism [6]. A cross-sectional study of high school students with metabolic syndrome found that reduced mtDNA content in peripheral white blood cells was associated with insulin resistance [7]. Similarly, a case-control study by Lee AH et al. demonstrated that mtDNA-CN in peripheral blood mononuclear cells was 1.28-fold lower in patients with MASLD compared to healthy controls [8]. These findings suggest that blood mtDNA-CN could serve as a simple and accessible biomarker for metabolic diseases such as fatty liver. However, most existing studies have been cross-sectional, making it difficult to establish causal relationships. Longitudinal studies are urgently needed to determine whether reduced mtDNA-CN contributes to disease progression or is merely a consequence of metabolic dysfunction.

In several animal studies, the relationship between mtDNA-CN and fatty liver has been validated. It has been reported that feeding male rats a 60% high-fat diet for more than 8 weeks significantly reduces hepatic mtDNA-CN [9, 10]. Another report showed that fatty liver induces a reduction in hepatic mtDNA-CN of approximately 50% in rats [11]. These findings highlight the potential of mtDNA-CN as a promising hepatic biomarker. However, significant challenges remain before mtDNA-CN can be practically applied. A key unresolved issue is whether changes in blood mtDNA-CN accurately reflect the physiological state of the liver [12]. Previous studies have primarily measured mtDNA levels within the liver, with limited investigation into the correlation between hepatic and blood mtDNA-CN levels. Addressing this gap requires a comprehensive analysis using animal models to clarify the relationship between mtDNA-CN in the liver and blood.

In this study, we combined longitudinal epidemiological analysis with animal models to comprehensively examine the impact of blood mtDNA-CN on the future development of SLD. In particular, we utilized animal models to elucidate the association between mtDNA levels in the blood and liver and to clarify the role of mtDNA-CN in the process of fatty liver development. These findings suggest that blood mtDNA-CN may serve as a novel and convenient predictive marker for SLD, contributing to the advancement of early prevention and diagnostic techniques for SLD.

## Methods

### Study participants: Population and sample selection

**Fig. 1:**
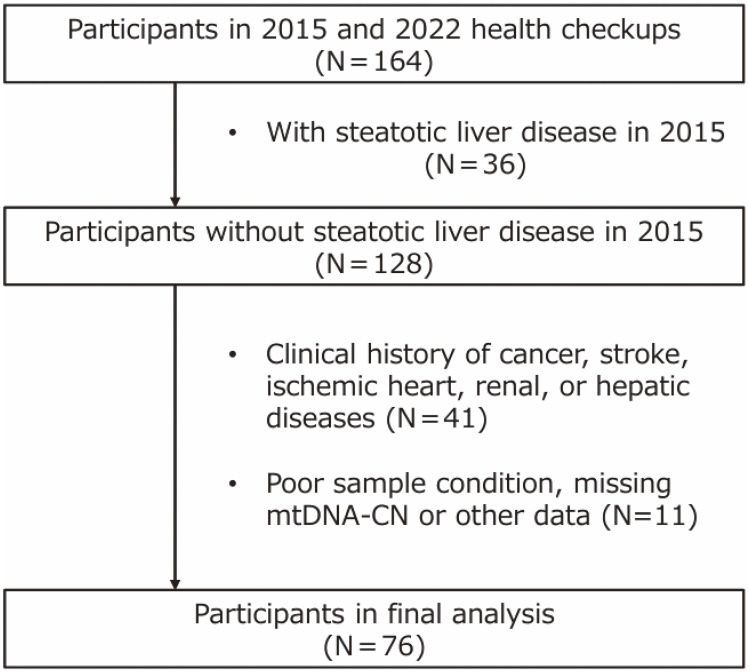
Flowchart of selected subjects for this study. This study was based on two health checkups conducted in 2015 and 2022, with 164 participants in both examinations. The study excluded 36 participants with SLD in 2015. Additionally, the study excluded 41 participants with a clinical history of cancer, stroke, ischemic heart, renal, or hepatic diseases and 11 participants with poor sample condition, missing mtDNA-CN or other data. Finally, 76 participants were included in this study. Abbreviations: SLD, steatotic liver disease; mtDNA-CN, mitochondrial DNA copy number.

This epidemiological study began in 1981 and included the study cohort from an annual, population-based health checkup in the town of Yakumo, Hokkaido, Japan. This human population of Yakumo Town was used in several previous epidemiological reports [13–15]. Every August, interdisciplinary research teams collaboratively conduct a survey in Yakumo Town, Hokkaido, to collect data and samples with the support of municipality staff. Briefly, a trained public health nurse measured each participant’s height, weight, and blood pressure and collected completed questionnaires and blood samples. The self-administered questionnaire included items regarding the participants’ sex, age, smoking habits (never; without a history, ever; with a history, or current), and alcohol consumption (never, ever, or current). Body mass index (BMI) was calculated by dividing weight (kg) by height squared (m^2^). The presence of intrahepatic steatosis was assessed by ultrasound examination using a ProSound α7 with a UST-9130 convex probe (Hitachi Aloka Medical, Tokyo, Japan) operated by three registered medical sonographers (Japan Society of Ultrasonics in Medicine) [16]. This study was based on two health examinations conducted in 2015 and 2022, with 164 participants (about 31.2% of the 2015 participants) in both examinations. Participants with fatty liver in 2015 (n = 36) were excluded, as were individuals with a clinical history of cancer, stroke, ischemic heart disease, renal disease, or hepatic disease (n = 41), and those with poor sample quality, missing mtDNA-CN data, or other incomplete datasets (n = 11). A total of 76 participants were included in the final analysis (Fig. 1). In addition, to determine the cut-off value for mtDNA-CN, 45 subjects with SLD as of 2015 and 180 healthy subjects matched for gender and age were selected from the 525 subjects who underwent health examinations in 2015 (Table 1). The study protocol was approved by the Ethics Committee of Fujita Health University (approval no. HG24-006).

**Table 1:** Characteristics of healthy and steatotic liver disease affected individuals in 2015.

	**Normal**	**SLD**	***P***
N	180	45	-
Age, years^a^	60.5 ± 9.9	60.6 ± 10.8	0.91
Male, n (%)^c^	86 (47.8)	26 (57.8)	0.25
BMI, kg/m^2 a^	22.4 ± 2.9	26.4 ± 3.0	<0.05
SBP, mmHg^a^	124.9 ± 20.6	135.4 ± 17.7	<0.05
HbA1c, %^a^	5.5 ± 0.28	6.0 ± 0.80	<0.05
TG, mg/dL^b^	78 (59–108)	144 (88–181)	<0.05
AST, IU/L^b^	21 (18–24)	26 (21–31)	<0.05
ALT, IU/L^b^	18 (14–23)	29 (24–40)	<0.05
γ-GTP, IU/L^b^	21 (14–32)	42 (28–56)	<0.05
LDL cholesterol, mg/dL	129 (106–149)	136 (118–150)	0.19
Smoking habit, n (%)^c^			
Never	83 (46.1)	16 (35.6)	0.39
Ever	65 (36.1)	18 (40.0)	
Current	32 (17.8)	11 (24.4)	
Alcohol consumption, n (%)^c^			
Never	78 (43.3)	23 (51.1)	0.52
Ever	1 (0.01)	0 (0)	
Current	101 (56.1)	22 (48.9)	

^a^Student *t* or ^b^Mann-Whitney U test were used for continuous variables and ^c^chi-square tests for categorical variables. Data of continuous variables are expressed as mean value ± standard deviation or median (25th–75th percentiles).

Abbreviations: SLD, steatotic liver disease; BMI, body mass index; SBP, systolic blood pressure; HbA1c, hemogrobinA1c; TG, triglyceride; AST, aspartate aminotransferase; ALT, alanine aminotransferase; γ-GTP, γ-glutamyl transferase.

### Animals

The experimental protocol was approved by the Animal Care and Use Committee at Fujita Health University (Permit No. H0862). 21-day-old male Sprague-Dawley rats (SLC, Shizuoka, Japan) were housed in environmentally controlled conditions at room temperature (23 °C ± 3 °C) under a 12:12 h light-dark cycle and *ad libitum* availability of standard laboratory rat chow (MF, Oriental Yeast Co., Ltd., Tokyo, Japan). After acclimatization for 1 week, the animals were divided into two experimental groups. The control group (CNT) received MF diet for 8 weeks, while the high fat diet group (HFD) received a 45% high fat diet (D12451, Research Diets, Inc., New Brunswick, NJ, USA) for the same period (n = 10–12/group). All animals always had *ad libitum* access to distilled water. Compositions of MF and high fat diet was showed as Supplemental Table 1. Body weight and food intake were recorded once per week. Access to food was withdrawn from all animals at 6 h before dissection. Animals were dissected at 2 and 8 weeks, and blood and liver samples were collected. We estimated analytical power given the sample size using G* power (version 3.1.9.7; Heinrich Heine Universität, Düsseldorf, Germany) to make sure there are no statistical problems.

### Histological analysis

The rat liver was processed in 4% formaldehyde for 24 h. The fixed liver was dehydrated with alcohol, embedded in paraffin, and sliced at 3 µm thickness using the Litratome REM-710 (Yamato Kohki, Saitama, Japan). Three slices were prepared for each liver at different depths. After deparaffinization and hydration, the slices were stained with Hematoxylin and eosin (H&E), and Sirius red. To analyze the area of lipid droplets in the sections stained with H&E, the white out area was quantitated by the ImageJ program using the 100× image in random microscopic fields from each animal. Similarly, to analyze the area of fibrosis in the sections stained with Sirius red, the red-stained area was quantitated.

### Cell culture and treatments

HepG2 (human hepatoma cell line) cells were cultured in D-MEM (high-glucose (4.5 g/L), Wako, Osaka, Japan) supplemented with 10% fetal bovine serum, 100 U/mL penicillin, and 0.1 mg/mL streptomycin [17]. Cultures were maintained in a humidified atmosphere of 5% CO_2_ at 37 °C. The cells were seeded into 24-well plates at a density of 1 × 10^5^ cells/mL and allowed to attach for 24 h. To investigate mitochondrial function and lipid accumulation, cells were treated with the following agents for 24 hours: ethidium bromide (EtBr; 0.5 or 2 µg/mL) [18], a mitochondrial DNA (mtDNA) replication inhibitor; rotenone (2 or 5 µM), an inhibitor of mitochondrial complex I; and oleic acid (100 µM) to promote lipid droplet formation.

### Oil red O staining

Oil Red O (Wako, Osaka, Japan) was dissolved in isopropanol to create a 0.3% stock solution. Oil red O stock solution and distilled water were mixed 3:2 and filtered through a sterile filter. HepG2 cells were processed in 4% formaldehyde for 1 h and stained with Oil Red O solution for 20 min. After washing once with 60% isopropanol and twice with distilled water, the cells were counterstained with Hematoxylin. To analyze the area of lipid droplets stained with Oil red O, the stain area was quantitated by the ImageJ program using the 400× image in random microscopic fields.

### Measurement of cell viability

Cellular viability was determined using Cell Counting Kit-8 (CCK-8, Wako, Osaka, Japan) according to manufacturer’s protocol. The cells were seeded into 96-well plates at a density of 1 × 10^5^ cells/mL and allowed to attach for 24 h. After chemical treatment, CCK-8 solution was added and incubated for 1 h at 37 °C. The absorbance was measured at 450 nm.

### Measurement of mitochondrial DNA copy number

Genomic DNA was extracted from rat or human EDTA-containing blood, rat liver tissue, and HepG2 cells using NucleoSpin Tissue kits (Takara, Shiga, Japan). The mtDNA-CN was measured using real-time polymerase chain reaction (PCR) as previously described [19], using QuantStudio™ 7 (Applied Biosystems, Foster City, CA, USA) and THUNDERBIRD Next SYBR qPCR mix (Toyobo, Osaka, Japan). The thermal conditions used were as follows: 95 °C for 1 minute, 40 cycles of 95 °C for 5 seconds, and 60 °C for 30 seconds. Relative quantification of mtDNA-CN was determined by the ratio of a mitochondrial genome DNA (mtDNA) and nuclear genome DNA (ncDNA). Actin-beta was used as the reference gene for calculating mtDNA-CN. The primers used for the target genes and calculated method for mtDNA-CN were reported earlier [20, 21].

### Statistical analysis

All data were statistically analyzed using EZR version 1.54 [22]. Continuous variables were compared using a student’s t-test, Mann-Whitney U test, or one-way analysis of variance. Data of continuous variables are expressed as mean value ± standard deviation or median (25th–75th percentiles). Categorical variables were compared using a chi-square test. Receiver operating characteristic (ROC) curve analysis of area under the curve (AUC) was performed to determine the cut-off value to predict fatty liver from mtDNA-CN of subjects in 2015. The cut-off values were adopted maximum value of calculated “sensitivity value - (1-specificity value)” as previously described [23]. From the cut-off values obtained, odds ratios (ORs) for new fatty liver were calculated for the two groups of 2022 subjects. ORs and 95% confidence intervals (CIs) for new fatty liver were calculated according to high levels of mtDNA-CN (≥cut-off value) as the reference. The model with age and sex as confounding factors was designated as adjusted model 1, and the model adjusted with BMI, smoking habit and alcohol consumption in addition to age and sex was designated as adjusted model 2. A *P*-value of <0.05 was considered statistically significant.

## Results

**Fig. 2:**
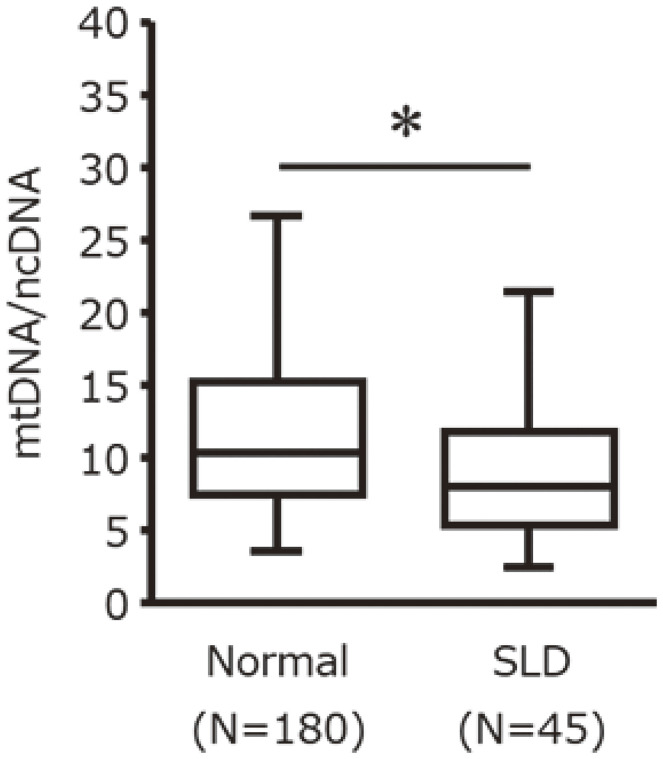
Comparison of blood mitochondrial DNA copy number in humans between normal and steatotic liver disease. Quantitative real-time PCR analysis blood mtDNA copy number in humans with SLD. The horizontal middle lines show the medians of the data, while the boxes show the 25th–75th percentiles (Q1 and Q3). The whiskers represent the maxima and minima. Student t-tests were performed to test for differences between the two groups. The statistical significance was set at P* < 0.05 compared with Normal. Abbreviations: PCR, Polymerase chain reaction; mtDNA-CN, mitochondrial DNA copy number; SLD, steatotic liver disease.

For the 2015 subjects shown in Table 1, we compared mtDNA-CN between the normal and SLD groups. Compared to the normal group, mtDNA-CN was significantly lower in the SLD group (Fig. 2, *P* < 0.05). ROC curve that differentiates fatty liver is drawn, the AUC and cut-off values were 0.65 and 6.64, respectively. The sensitivity and specificity were 0.40 and 0.85, respectively.

The characteristics of the subjects of this study, selected by the flow chart in Fig. 1, are shown in Table 2. Based on the mtDNA-CN cut-off value determined in Fig. 2, participants were classified into low and high mtDNA-CN groups. There were no significant differences between the two groups in all categories, including age, sex, BMI, liver function, and lifestyle. This indicates that mtDNA-CN differences are not confounded by baseline characteristics and may reflect an intrinsic association with SLD.

**Table 2:** Characteristics of participants followed from 2015 to 2022.

	**mtDNA-CN**		***P***

	**Low (<6.64)**	**High (6.64≤)**	
N	17	59	-
Age, years^a^	61.6 ± 8.7	60.4 ± 8.4	0.62
Male, n (%)^c^	7 (41.2)	22 (37.3)	0.78
BMI, kg/m^2 a^	22.8 ± 3.7	22.8 ± 3.0	0.98
SBP, mmHg^a^	128.4 ± 18.6	122.6 ± 18.2	0.26
HbA1c, %^a^	5.7 ± 0.36	5.7 ± 0.43	0.80
TG, mg/dL ^b^	68 (52–100)	78 (59–110)	0.36
AST, IU/L^b^	21 (17–24)	21 (19–24)	0.64
ALT, IU/L^b^	17 (15–23)	19 (16–24)	0.40
γ-GTP, IU/L^b^	27 (15–36)	21 (14–33)	0.31
LDL cholesterol, mg/dL	122 (107–143)	117 (101–146)	0.77
Smoking habit, n (%)^c^			
Never	10 (58.8)	31 (52.5)	0.79
Ever	5 (29.4)	17 (28.8)	
Current	2 (11.8)	11 (18.6)	
Alcohol consumption, n (%)^c^			
Never	9 (52.9)	30 (50.8)	0.74
Ever	0 (0)	2 (0.03)	
Current	8 (47.1)	27 (45.8)	
SLD in 2022, n (%)^c^			
No	10 (58.8)	47 (79.7)	0.15
Yes	7 (41.2)	12 (20.3)	

^a^Student *t* or ^b^Mann-Whitney U test were used for continuous variables and ^c^chi-square tests for categorical variables. Data of continuous variables are expressed as mean value ± standard deviation or median (25th–75th percentiles).

Abbreviations: mtDNA-CN, mitochondrial DNA copy number; BMI, body mass index; SBP, systolic blood pressure; HbA1c, hemogrobinA1c; TG, triglyceride; AST, aspartate aminotransferase; ALT, alanine aminotransferase; γ-GTP, γ-glutamyl transferase; SLD, steatotic liver disease.

The ORs and 95% CIs were calculated to determine the effect of low mtDNA-CN on fatty liver diseases, using high mtDNA-CN as the reference (Table 3). A trend toward a higher risk of SLD was observed with lower mtDNA-CN values (ORs [95% CIs]; 2.74 [0.86–8.71], *P* = 0.09). A similar trend was observed in adjustment model 1 (ORs [95% CIs]; 2.78 [0.84–9.21], *P* = 0.10). In adjusted model 2, lower levels of mtDNA-CN were associated with significantly higher SLD risk (ORs [95% CIs]; 4.93 [1.08–22.50], *P* = 0.04).

**Table 3:** ORs and 95%CI for steatotic liver disease development according to mtDNA-CN.

**mtDNA-CN** **Cut-off**	**SLD** **n (%)**	**Crude**		**Adjusted model 1**		**Adjusted model 2**	
		
		**ORs (95%CI)**	***P***	**ORs (95%CI)**	***P***	**ORs (95%CI)**	***P***
Low (<6.64)	7 (41.2)	2.74 (0.86–8.71)	0.09	2.78 (0.84–9.21)	0.10	4.93 (1.08–22.50)	0.04
High (6.64≤)	12 (20.3)	1.00	-	1.00	-	1.00	-

Adjusted model 1; adjusted for age and sex.

Adjusted model 2; adjusted for age, sex, BMI, smoking habit and alcohol consumption.

Abbreviations: ORs, odds ratios; CI, confidence interval; SLD, steatotic liver disease; mtDNA-CN, mitochondrial DNA copy number; BMI, body mass index.

**Fig. 3:**
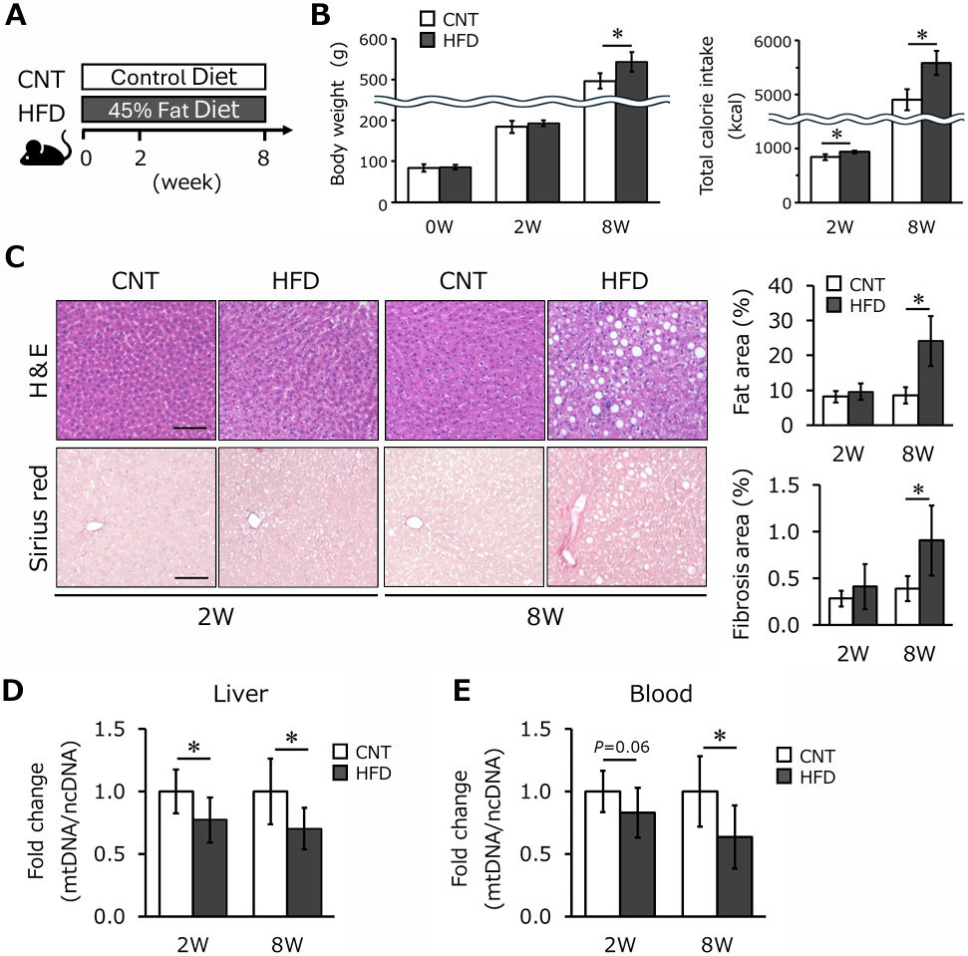
Analysis of phenotype and mitochondrial DNA copy number in rat. Rats were fed a 45% high-fat diet and dissected at 2 and 8 weeks (A). Body weight and total calorie intake at 2 and 8 weeks in rats (B). H&E and Sirius red stained images of rat liver tissue at 2 and 8 weeks (C). Scale bar 100 µm. Quantitative real-time PCR analysis of hepatic (D) and blood (E) mtDNA copy number at 2 and 8 weeks in rats. Results are shown relative to CNT for each week. All values are presented as means ± standard deviation (n = 5–6/group). The statistical significance was set at P* < 0.05 compared with CNT for each week. Abbreviations: H&E, Hematoxylin and eosin; PCR, Polymerase chain reaction; mtDNA-CN, mitochondrial DNA copy number; CNT, control group; HFD, high fat diet group.

In the animal model, rats were fed a high-fat diet for 8 weeks to induce fatty liver (Fig. 3A). No significant difference in body weight was observed between groups at 2 weeks, but total calorie intake was significantly higher in the HFD group, and body weight became significantly higher in the HFD group by week 8 (Fig. 3B, *P* < 0.05). Histological examination revealed no significant differences in lipid and fibrosis areas at 2 weeks; however, both were significantly increased in the HFD group by week 8 (Fig. 3C, *P* < 0.05). These results suggest that an 8-week HFD induces fatty liver development, with progression occurring between weeks 2 and 8. Consistent with the findings in humans, mtDNA-CN in the liver was significantly decreased in the HFD group at both time points (Fig. 3D, *P* < 0.05). Similarly, blood mtDNA-CN showed a decreasing trend in the HFD group at 2 weeks (*P* = 0.06) and became significantly lower by 8 weeks (Fig. 3E, *P* < 0.05).

**Fig. 4:**
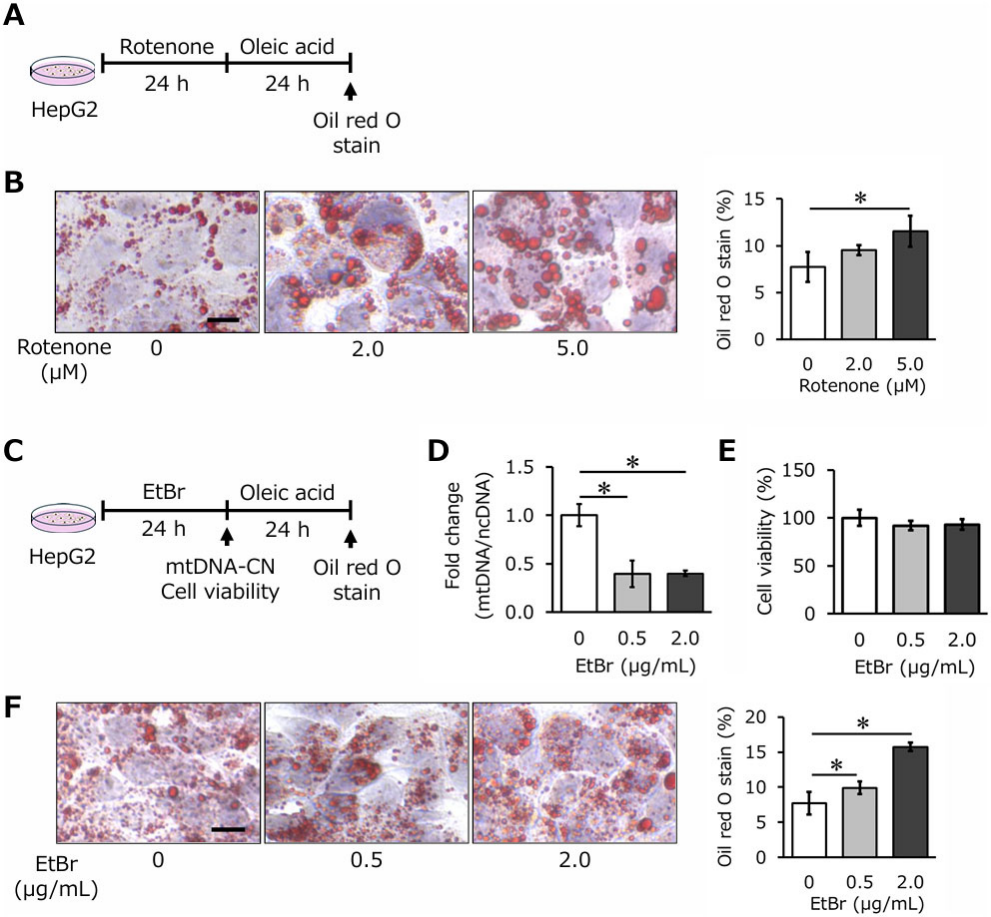
Oleic acid uptake during reduced mitochondrial function and mitochondrial DNA copy number. Rotenone (0, 2.0, 5.0 µM) and oleic acid (100 µM) were exposed to HepG2 for 24 h each (A). Oil red stained image of HepG2 cells exposed to rotenone followed by oleic acid (B). Scale bar 10 µm. EtBr (0, 0.5, 2.0 µg/mL) and oleic acid (100 µM) were exposed to HepG2 for 24 h each (C). Quantitative real-time PCR analysis of hepatic mtDNA-CN after 24 h exposure to EtBr (D). Results are shown relative to untreated cells. Cell viability of HepG2 cells after 24 h exposure to EtBr (E). Oil red stained image of HepG2 cells exposed to EtBr followed by oleic acid (F). Scale bar 10 µm. All values are presented as means ± standard deviation (n = 3/group). The statistical significance was set at P* < 0.05 compared with untreated cells. Abbreviations: mtDNA-CN, mitochondrial DNA copy number; EtBr, ethidium bromide.

To further explore the link between mitochondrial dysfunction and fat accumulation, HepG2 cells were treated with rotenone, an inhibitor of mitochondrial complex I. Oleic acid was added to induce lipid accumulation (Fig. 4A). Oil Red O staining revealed that fat accumulation was markedly enhanced in HepG2 cells with rotenone-induced mitochondrial dysfunction (Fig. 4B). Similarly, mtDNA-CN reduction was induced by ethidium bromide (EtBr) treatment, leading to significant mtDNA-CN depletion without affecting cell viability (Fig. 4C–E, *P* < 0.05). In these cells, fat accumulation was also markedly increased (Fig. 4F). These cell-based findings are consistent with the human and animal data, demonstrating that mtDNA-CN reduction and mitochondrial dysfunction are closely linked to lipid accumulation.

## Discussion

This study employed a multifaceted approach, integrating longitudinal epidemiological analysis, animal models, and in vitro experiments, to elucidate the role of mtDNA-CN in the development of SLD. For the first time, we demonstrated through a longitudinal cohort study that lower blood mtDNA-CN levels are associated with an increased risk of future SLD development. The epidemiological findings revealed that individuals with lower mtDNA-CN had a significantly higher risk of developing SLD, even after adjusting for potential confounders such as BMI and smoking habits. These results were complemented by experimental evidence from animal models and HepG2 cells, which consistently demonstrated a relationship between mtDNA-CN reduction, mitochondrial dysfunction, and lipid accumulation. These results not only advance our understanding of the role of mitochondrial dysfunction in SLD pathogenesis but also open new avenues for early detection and targeted interventions.

The findings of this study underscore the critical role of mitochondrial dysfunction, as indicated by mtDNA-CN reduction, in the pathogenesis of SLD. Mitochondria are essential for hepatic lipid metabolism, including β-oxidation of fatty acids, oxidative phosphorylation, and regulation of oxidative stress [24]. A reduction in mtDNA-CN likely impairs mitochondrial biogenesis and function, leading to deficits in energy metabolism, increased production of reactive oxygen species (ROS), and lipid accumulation in hepatocytes. These mechanistic changes align with the observed association between lower mtDNA-CN levels and an elevated risk of SLD development in both human and animal models. In animal studies, mtDNA-CN reductions were observed in both liver and blood during fatty liver progression, suggesting a systemic impact of mitochondrial dysfunction. This systemic observation reinforces the potential utility of blood mtDNA-CN as a non-invasive biomarker for monitoring SLD. Furthermore, the in vitro experiments demonstrated that artificially induced mtDNA-CN depletion through ethidium bromide or mitochondrial dysfunction via rotenone treatment directly enhanced lipid accumulation in HepG2 cells. These findings provide strong mechanistic support for the epidemiological observations and emphasize the biological plausibility of mtDNA-CN serving as a key player in SLD pathophysiology.

Previous studies have reported an association between blood mtDNA-CN and SLD or metabolic disorders, but these were primarily cross-sectional, limiting their ability to establish causal relationships [7, 8]. In contrast, this study employed a longitudinal design and demonstrated that reductions in blood mtDNA-CN may precede the onset of SLD. Additionally, animal models showed that reductions in hepatic mtDNA-CN are associated with the progression of SLD [9, 10]. Importantly, this study is the first to evaluate blood mtDNA-CN in an animal model, revealing parallel reductions in both blood and hepatic mtDNA-CN in rats fed a high-fat diet. This parallel reduction supports the hypothesis that blood mtDNA-CN reflects liver pathology and aligns with epidemiological findings of decreased blood mtDNA-CN in patients with SLD [8]. These results suggest that changes in hepatic mtDNA-CN may directly or indirectly influence blood mtDNA-CN, potentially through systemic mitochondrial dynamics or the release of mtDNA fragments into the circulation. Together, these findings highlight the potential of blood mtDNA-CN as a reliable and non-invasive biomarker for liver health and SLD prediction.

The reduction of mtDNA-CN may affect the expression levels of a group of genes encoded by mtDNA [25]. In the present study, the expression of mtDNA-coding genes (*ND6*, *Cytb* and *ATP6*) present in respiratory chain complexes I, III and V is significantly reduced in HFD (Supplemental Fig. 1). This phenomenon could be caused by decreasing mtDNA content. Since these genes are closely linked to mitochondrial metabolic functions, their downregulation could lead to disruptions in energy metabolism. Supporting this, fat accumulation was enhanced when mitochondrial function was reduced by rotenone loading of HepG2 cells. Also, HepG2 cells with low mtDNA-CN showed increased fat uptake, providing further evidence of a potential link between mtDNA-CN and lipid metabolism. Collectively, it is suggested that a high-fat diet can derail lipid metabolism by reducing the master proteins involved in the maintenance of mtDNA, leading to a reduction in copy number, and also by reducing the function of the electron transfer system. These results may aid interpretation of the relationship between mtDNA-CN and metabolic diseases such as SLD.

In this study, gene expression of *mitochondrial transcription factor A* (*Tfam*) in the liver was reduced after an 8-week high-fat diet (Supplemental Fig. 1). This phenomenon was supported by previous findings that Tfam and peroxisome proliferator-activated receptor-gamma coactivator 1 alpha (Pgc1a), master proteins that are involved in mtDNA biogenesis, are reduced in the liver of rats by a 15-week high-fat diet [26]. These findings suggest that the decrease in mtDNA-CN due to high-fat diet intake is due to reduced expression of Tfam and Pgc1a. Moreover, gene expression of these two proteins has been reported to be subject to epigenetic gene regulation, such as DNA methylation [27–29]. Furthermore, epigenetic gene regulation is altered by factors such as diet [30–32] and has been reported to be associated with metabolic disorders, including fatty liver [33–35]. Based on these findings, it is considered that high-fat diet intake reduces mtDNA-CN by inducing DNA methylation abnormalities, leading to suppressed expression of Tfam and Pgc1a. Further detailed mechanistic elucidation is needed to explain this hypothesis.

We consider the relationship between mtDNA-CN and liver fat to be complex and potentially bidirectional. In humans, lower mtDNA-CN levels may reflect an underlying genetic predisposition or accumulated mitochondrial dysfunction due to metabolic or environmental stressors. However, our findings from the animal and cell models provide additional mechanistic insight. In the animal model, a high-fat diet exposure led to a significant reduction in hepatic mtDNA-CN, accompanied by liver fat accumulation. In vitro, direct reduction of mtDNA-CN via EtBr treatment induced fat accumulation in HepG2 cells without reducing cell viability. These results suggest that reduced mtDNA-CN can act as a mediator, not merely a marker, of steatotic liver development in response to environmental stimuli. Thus, we propose that mtDNA-CN may serve both as a sensitive indicator of mitochondrial health and as a potential mechanistic link between environmental exposures (e.g., high-fat diet) and liver fat accumulation.

So far, we have compared human and rat mtDNA as if they were the same. Mitochondria originate from a common eukaryotic ancestor and have retained a high degree of evolutionary conservation across species. Comparative studies have demonstrated that the mitochondrial genome structure, replication mechanisms, and encoded protein functions are largely conserved between humans and mice [36, 37]. Consequently, mtDNA-CN is generally regarded as a functionally equivalent indicator of mitochondrial status in both species, but they differ slightly in genome size and sequence. However, the fact that human and rodent mitochondrial genomes differ slightly in size and sequence will require careful comparison and evaluation.

This study has several limitations. First, the epidemiological analysis was constrained by a relatively small sample size, as the number of participants diagnosed with SLD between 2015 and 2022 was limited. This limitation may have reduced the statistical power to detect subtle associations and limits the generalizability of the findings to other populations. Future studies with larger and more diverse cohorts are needed to validate these results and confirm the predictive utility of mtDNA-CN for SLD. Second, reductions in mtDNA-CN have been observed in other organs, such as the testes or muscle tissue [38–40], suggesting that a high-fat diet may lead to widespread declines in mtDNA-CN across multiple tissues. These findings highlight the systemic impact of mitochondrial dysfunction and the potential of blood mtDNA-CN as a marker not only of liver health but also of overall mitochondrial status. However, further studies are needed to clarify how blood mtDNA-CN reflects mitochondrial function across different organs. Third, the animal studies conducted in this study could not track changes in mtDNA-CN over time due to high-fat diet intake. In the future, the experimental protocol should be reconsidered, and these points should be mentioned. Another point, in this study, we used HepG2 to clarify the pathophysiology of mtDNA-CN and SLD in humans. For more detailed evaluation, it is preferable to use primary cultured cells, organoids etc. instead of cell lines [17]. Finally, while this study demonstrates the potential of mtDNA-CN as a predictive biomarker, the clinical application of these findings remains to be established. Validation in larger cohorts and real-world healthcare settings is essential to assess the feasibility, sensitivity, and specificity of using blood mtDNA-CN for the early detection and monitoring of fatty liver disease.

In conclusion, reduced blood mtDNA-CN may be associated with the future development of SLD. This study suggests that mtDNA-CN might serve as a biomarker for early detection and risk assessment. Mitochondrial DNA is highly stable, and its extraction uses the same protocols and reagents as those employed for nuclear DNA, allowing for automation and high-throughput processing. The quantification of mtDNA-CN can be performed using standard real-time PCR platforms, which are widely available in clinical and research laboratories. As such, the cost per sample is relatively low, and the workflow is simple and scalable. While further validation in broader clinical settings is warranted, we believe that the measurement of mtDNA-CN is both technically feasible and cost-effective, making it a promising candidate for future clinical applications.

## Abbreviations

mtDNA: Mitochondrial DNA
mtDNA-CN: Mitochondrial DNA-copy number
SLD: Steatotic liver disease
MASLD: Metabolic dysfunction-associated steatotic liver disease
EtBr: Ethidium bromide
BMI: Body mass index
EDTA: Ethylenediaminetetraacetic acid
PCR: Polymerase chain reaction
ROC: Receiver operating characteristic
AUC: Area under the curve
CIs: Confidence intervals
SBP: Systolic blood pressure
HbA1c: HemogrobinA1c
TG: Triglyceride
AST: Aspartate aminotransferase
ALT: Alanine aminotransferase
γ-GTP: γ-glutamyl transferase

## Supplementary information

**Additional file 1:** **Supplemental table 1.** A type of diet and Ingredient composition in this study. **Supplemental Figure 1.** Comparison of messenger RNA expression levels during 8 weeks of high-fat diet intake in rats.

## Declarations

### Availability of data and material

The datasets analyzed during the current study are available from the corresponding author on reasonable request.
