# Supplementary material for: Peripheral blood mitochondrial DNA copy number as a predictor of steatotic liver disease development: insights from epidemiological and experimental studies
Source: Environ Health Prev Med. 2025 May 28;30:42. doi: 10.1265/ehpm.25-00025 (PMC12127083; doi:10.1265/ehpm.25-00025)
Supplement: Supplementary file 1 — Additional file 1: Supplemental table 1. A type of diet and Ingredient composition in this study. Supplemental Figure 1. Comparison of messenger RNA expression levels during 8 weeks of high-fat diet intake in rats. [file ehpm-30-042-s001.docx]

**Peripheral Blood Mitochondrial DNA Copy Number as a Predictor of Steatotic Liver Disease Development: Insights from Epidemiological and Experimental Studies**

Genki Mizuno, Atsushi Teshigawara, Hiroya Yamada, Eiji Munetsuna, Yoshiki Tsuboi, Yuji Hattori, Mirai Yamazaki, Yoshitaka Ando, Itsuki Kageyama, Takuya Wakasugi, Naohiro Ichino, Keisuke Osakabe, Keiko Sugimoto, Ryosuke Fujii, Hiroaki Ishikawa, Nobutaka Ohgami, Koji Ohashi, Koji Suzuki

| **Supplemental table 1. A type of diet and Ingredient composition in this study.** | | |
| --- | --- | --- |
| Group | CNT | HFD |
| Diet | MF | D12451 |
| Calorie (kcal/g) | 3.59 | 4.73 |
| Ingredients (g/kg) | Sucrose (500) | Sucrose (172.8) |
|  | Casein (200) | Casein (200) |
|  | Corn starch (150) | Corn starch (72.8) |
|  | Mineral Mix (35) | Mineral Mix (10) |
|  | Vitamin Mix (10) | Vitamin Mix (10) |
|  | Choline bitartrate (2) | Choline bitartrate (2) |
|  | Corn oil (50) | Soybean oil (25) |
|  | DL-methionine (3) | L-Cystine (3) |
|  |  | Maltodextrin 10 (100) |
|  |  | Cellulose (50) |
|  |  | Lard (177.5) |
|  |  | DiCalcium Phosphate (13) |
|  |  | Calcium Carbonate (5.5) |
|  |  | Potassium Citrate (16.5) |
|  |  | FD＆C Red Dye #40 (0.05) |


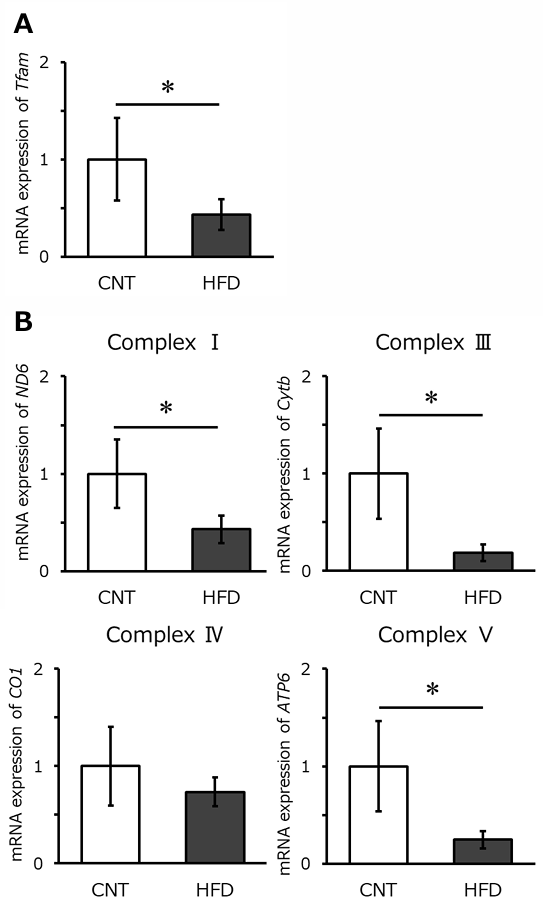


**Supplemental Figure 1. Comparison of messenger RNA expression levels during 8 weeks of high-fat diet intake in rats.**

Quantitative real-time PCR analysis of hepatic mRNA expression of *Tfam* at 8 weeks in rats (A). Quantitative real-time PCR analysis of hepatic mRNA expression of mtDNA-coding genes (*ND6, Cytb,* *CO1,* and *ATP6*) present in respiratory chain complexes I, III, Ⅳ and V at 8 weeks in rats (B). Total RNA was isolated from the rats’ livers using TRIzol reagent (Invitrogen, Carlsbad, CA, USA), according to the manufacturer's instructions. Complementary DNA was prepared using M-MLV reverse transcriptase (Invitrogen) in conjunction with a random primer mix (Takara, Shiga, Japan). Real-time PCR results were normalized to the expression levels of the housekeeping gene β-ACTIN, and ΔΔCt values were calculated for each gene. Results are shown relative to CNT. Values are presented as means ± standard deviation (n=5-6/group). The statistical significance was set at *P**< 0.05 compared with CNT.

Abbreviations: PCR, Polymerase chain reaction; mRNA, messenger RNA; Tfam, mitochondrial transcription factor A; CNT, control group; HFD, high fat diet group.
